# Efficacy and Safety of Leadless Pacemakers for Atrioventricular Synchronous Pacing: A Systematic Review and Meta-Analysis

**DOI:** 10.3390/jcm12072512

**Published:** 2023-03-27

**Authors:** Sijin Wu, Yuanhao Jin, Wenzhao Lu, Zhongli Chen, Yan Dai, Keping Chen

**Affiliations:** State Key Laboratory of Cardiovascular Disease, Arrhythmia Center, National Center for Cardiovascular Diseases, Fuwai Hospital, Chinese Academy of Medical Sciences & Peking Union Medical College, Beijing 100037, China

**Keywords:** leadless pacemaker, Micra, atrioventricular synchronous pacing, atrioventricular synchrony, meta-analysis

## Abstract

Leadless pacemakers with an atrioventricular synchrony algorithm represent a novel technology for patients qualified for VDD pacing. The current evidence of their performance is limited to several small-scale observational studies. This systematic review and meta-analysis aimed to evaluate the efficacy and safety of this new technology. We systematically searched the PubMed, Embase, and Cochrane library databases from their inception to 12 September 2022. The primary efficacy outcome was atrioventricular synchrony after implantation, whereas the secondary efficacy outcome was the change in cardiac output represented by the left ventricular outflow tract velocity time integral (LVOT-VTI). The primary safety outcome was major complications related to the procedures and the algorithm. Means or mean differences with 95% confidence interval (95% CI) were combined using a random-effects model or a fixed-effects model. Finally, 8 published studies with 464 participants were included in the qualitative analysis. The pooled atrioventricular synchrony proportion was 78.9% (95% CI 71.9–86.0%), and a further meta-regression did not screen factors that contributed significantly to the heterogeneity. Additionally, a significant increase in atrioventricular synchrony of 11.3% (95% CI 7.0–15.7%, *p* < 0.01) was achieved in patients experiencing programming optimization. LVOT-VTI was significantly increased by 1.9 cm (95% CI 1.2–2.6, *p* < 0.01), compared with the VVI pacing mode. The overall incidence of complications was approximately 6.3%, with major complications related to the algorithm being extremely low. Overall, leadless pacemakers with atrioventricular synchronous pacing demonstrated favorable safety and efficacy. Future data on their long-term performance are required to facilitate their widespread adoption in clinical practice.

## 1. Introduction

Leadless pacemakers have been developed to overcome the lead- and pocket-related adverse effects of conventional transvenous pacemakers. Because of the absence of leads and pulse generator pockets, patients with implanted leadless pacemakers will not incur major complications as those with conventional transvenous pacemakers, including lead failure and dislodgement, pocket hematomas, pneumothorax, and infection, which affect as many as 12.6% of patients who receive transvenous pacemakers [1,2]. Accumulated evidence has revealed that leadless pacemakers could achieve a satisfactory electrical performance and contribute to a nearly 50–60% reduction in complications compared to transvenous pacemakers [3,4,5]. Nowadays, leadless pacemakers have been increasingly recommended for patients requiring single-chamber ventricular pacing. Nevertheless, until recently, their use was restricted to a small percentage of pacemaker implantations due to their inherent single-chamber design.

In recent years, a new generation of leadless pacemakers has emerged, which features an accelerometer-based atrioventricular synchrony algorithm. The device can track mechanical atrial contraction and provide atrioventricular synchronous pacing when it is programmed with a VDD pacing mode. Atrial sensing is accomplished by detecting the atrial contraction through an integrated accelerometer, which utilizes either a single-axis accelerometer vector or a combination of vectors. A preliminary proof-of-concept study demonstrated that Micra single-chamber leadless pacemakers with the synchrony algorithm were feasible and significantly facilitated atrioventricular synchrony in patients with an atrioventricular block [6]. The MARVEL 2 (Micra Atrial tRacking using a Ventricular accelerometer 2) study reported an enhanced algorithm that could increase the median atrioventricular synchrony from 27% to 94% and improve the cardiac output when comparing the VVI pacing mode with the VDD pacing mode [7]. 

Based on its favorable performance, the Micra^TM^ system with atrioventricular synchrony (Micra-AV, Medtronic, Minneapolis, MN, USA) was approved by the United States Food and Drug Administration in 2020. This approval substantially broadened the spectrum of patients eligible for leadless pacing. Subsequently, several observational studies reported on the early experience with this novel technology in patients with different pacing indications, including atrioventricular block, sinus node dysfunction, and atrial fibrillation with bradycardia [8,9,10]. However, the results of these studies varied. For instance, in a real-world study, the atrioventricular synchrony was much lower than in the initial MARVEL 2 study (62.9% vs 94.3%) [9]. Nevertheless, conclusions are limited due to the small sample sizes. The effects of this new-generation leadless pacemaker on atrioventricular synchrony and its potential complications remain unclear. 

To date, there has been no systematic review and meta-analysis of leadless atrioventricular synchronous pacing. Therefore, we sought to conduct a systematic review and meta-analysis of the published studies to better investigate the safety and efficacy of this new technology.

## 2. Methods 

This study was performed in accordance with the PRISMA (Preferred Reporting Items for Systematic Reviews and Meta-Analyses) statement [11]. Before starting the literature search, we prospectively registered the study protocol in the international prospective register of systematic reviews (PROSPERO registration number: CRD42022361968).

### 2.1. Data Sources and Searches

A systematic literature search was performed on 13 September 2022 by searching the medical databases PubMed, Embase, and Cochrane library. The search strategy included the following terms: (“leadless”, “wireless”, or “Micra”) and (“synchron*”, “atrioventricular”, or “AV”) and (“pacemaker” or “pacing” or “artificial pacemaker”). All databases were searched from inception up to 12 September 2022, without language restrictions. A detailed search algorithm is available in Supplementary Method S1.

### 2.2. Inclusion and Exclusion Criteria

Two investigators (S.W. and W.L.) separately carried out the study selection according to the predefined inclusion and exclusion criteria. Discrepancies were resolved by consensus or discussion with other investigators. All published clinical studies that involved the use of atrioventricular synchronous leadless pacemakers and reported atrioventricular synchrony were considered eligible for our study. Exclusion criteria were set as followed: (a) reviews, comments, conference abstracts, case reports, non-human studies, or other irrelevant studies; (b) studies related to leadless pacemakers without reporting atrioventricular synchrony; (c) studies related to leadless pacemakers combining other interventions (e.g., ablation, defibrillation, or resynchronization); (d) studies on the same population or sub-analyses of another included study.

### 2.3. Outcome Measures

The primary efficacy outcome was specified as atrioventricular synchrony following the implantation of atrioventricular synchronous leadless pacemakers. Due to the variability of the atrioventricular synchrony measurements, we considered the atrioventricular synchrony proportion to be the most precise when measured in patients with sinus rates of 50–80/min and predominantly paced. The secondary efficacy outcome was the change in cardiac output compared between VVI and VDD pacing modes. In some studies, the cardiac output was represented by the left ventricular outflow tract velocity time integral (LVOT-VTI), a proxy of left ventricular stroke volume, which was measured using echocardiography. The primary safety outcome was major complications related to both the atrioventricular synchrony algorithm and the procedures. Especially, complications related to the synchrony algorithm were defined as ventricular pauses and oversensing-induced tachycardia.

### 2.4. Data Extraction and Quality Assessments

The data of the included studies were extracted by two independent reviewers (S.W. and W.L.) using a prespecified data extraction electronic form and were checked for accuracy by the senior author (K.C.). The retrieved data included authors, year of publication, study design, population size (enrollment and efficacy evaluation), follow-up duration, patient demographics (age, sex, and indication for pacing), the definition of atrioventricular synchrony, atrioventricular synchrony proportion, LVOT-VTI in both VVI and VDD modes, and major complications. In studies reporting multiple atrioventricular synchrony proportions with multiple follow-up visits, we extracted the atrioventricular synchrony values of the first follow-up visit. For studies that had subgroups experiencing optimized programming, we extracted the size of the subgroup, the main parameters for optimized programming, and the baseline and the optimized atrioventricular synchrony proportion.

The quality of the included studies was assessed using the National Institutes of Health quality assessment tool for before–after (pre–post) studies with no control group [12]. On this basis, the methodological quality of each study was assessed as good, fair, or poor (Supplementary Table S1). 

### 2.5. Data Synthesis and Analysis

All continuous variables are presented as mean ± standard deviation (SD). For studies that reported the continuous variables as median (interquartile range) or mean with a 95% confidence interval (95%CI), we estimated and transformed the values to mean ± SD using the optimal methods according to the Cochrane Handbook for data conversion [13]. The estimated data were then combined. For the outcome variables, the pooled atrioventricular synchrony proportion is presented as mean with 95%CI, since it had no control group, and the combined LVOT-VTI are reported as mean differences with 95%CI to describe the change values. Heterogeneity was assessed by the Cochrane Q test and *I*^2^ statistics. The pooled results were combined using a random-effects model when heterogeneity was high (*I*^2^ > 50% or *p* value <0.1 for the Q test), otherwise a fixed-effects model was chosen, and forest plots were constructed. In cases where heterogeneity was detected, we performed a univariable meta-regression using the residual maximum likelihood method of baseline variables to screen for factors contributing to the heterogeneity. The funnel plot and Egger’s test of the included studies were examined to assess potential publication biases. We conducted additional sensitivity analyses by iteratively omitting each eligible trial to account for different types of emerging bias. All data analyses were performed using R (version 4.1.2, 1 November 2021) and the “meta” package in R (version 5.2-0). Statistical significance was considered at a 2-tailed *p* value < 0.05.

## 3. Results

### 3.1. Study and Patient Characteristics

**Figure 1:**
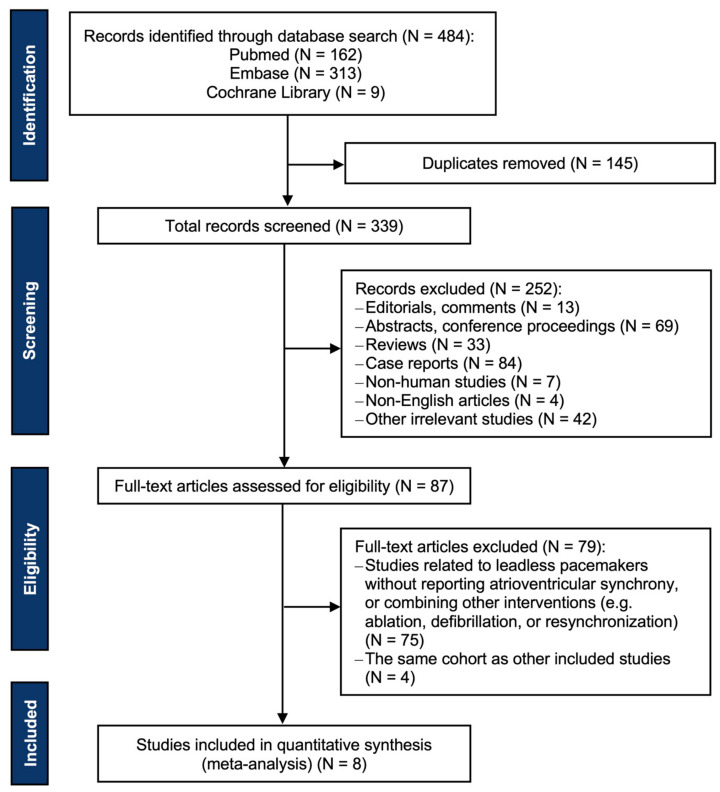
PRISMA flow diagram of the literature search.

Overall, 484 records were identified through database search, of which 397 were excluded after removing duplicates and screening titles and abstracts. Eighty-seven full-text articles were assessed for eligibility. Of these articles, 75 studies related to leadless pacemakers were excluded because they did not report atrioventricular synchrony or combined other interventions, and 4 studies because they used the same cohort as other included studies. Ultimately, eight studies met the inclusion criteria for the meta-analysis [6,7,8,9,10,14,15,16]. The PRISMA flow chart is displayed in Figure 1. All eight studies had a single-arm observational design: three were multicenter prospective studies, three were single-center prospective studies, and two were single-center retrospective studies. Of the eight studies, five were evaluated as having good quality, whereas three were graded as fair. The study characteristics are listed in Table 1.

A total of 464 participants were included, with a mean age of 76.3 ± 4.2 years and a female proportion of 43.8%. The implanted devices were all Micra-AV. The indications for Micra-AV implantation included both atrioventricular block and sinus node dysfunction with or without atrial fibrillation. One study enrolled patients undergoing transcatheter aortic valve implantation [16]. Four studies reported pacing burden, which was represented as percentage of ventricular pacing (VP%). Among them, the AccelAV study reported a VP% of almost 100%, as it was analyzed only in patients with a complete atrioventricular block. The median VP% reported by Arps et al. was just 10%, since this study included patients with sinus node dysfunction and intrinsic atrioventricular conduction. The numerical differences in pacing burden among different studies may be related to different patient indications. The baseline patient characteristics are summarized in Table 2. 

### 3.2. Atrioventricular Synchrony

All eight studies reported atrioventricular synchrony following the implantation of atrioventricular synchronous leadless pacemakers, but the definition of atrioventricular synchrony varied across the studies. Most commonly, atrioventricular synchrony was defined as a *p* wave visible on surface electrocardiography followed by a ventricular event <300 ms. Other studies defined it as “atrial mechanical sensed–ventricular pacing”, which referred to the percentage of ventricular pacing preceded by a detected atrial mechanical event. 

**Figure 2:**
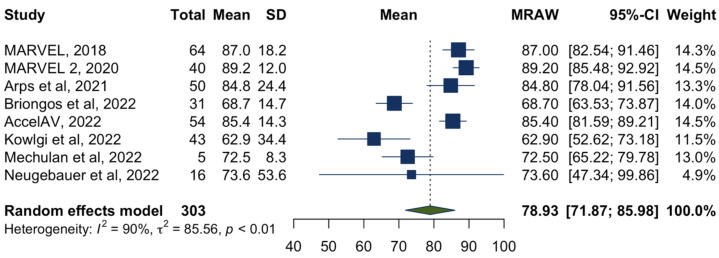
Forest plot of the atrioventricular synchrony proportion (%) [6,7,8,9,10,14,15,16].

A total of 303 participants who had the synchrony algorithm downloaded to their pacemakers were included for primary efficacy evaluation. The average atrioventricular synchrony proportion ranged from 62.9% to 89.2% across the studies, with a pooled atrioventricular synchrony proportion of 78.9% (95%CI 71.9–86.0%), as shown in the forest plot (Figure 2). However, there was high heterogeneity between studies (*I*^2^ = 90%, *p* < 0.01). Thus, we performed a further meta-regression using the following covariates: age, gender, indications for leadless atrioventricular synchronous pacing, study quality, and whether the patients underwent Micra-AV implantation following transcatheter aortic valve implantation. None of these variables significantly contributed to the observed heterogeneity (*p* = 0.23, *p* = 0.86, *p* = 0.59, *p* = 0.27, and *p* = 0.51, respectively). 

**Figure 3:**
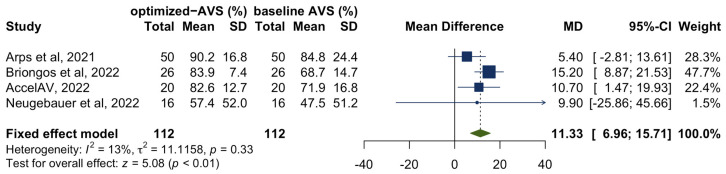
Forest plot of the change in atrioventricular synchrony after programming optimization. Abbreviation: AVS = atrioventricular synchrony [8,10,14,15].

Additionally, 4 studies [8,10,14,15] also explored the impact of manually optimized reprogramming on atrioventricular synchrony, compromising a total of 112 patients. A meta-analysis was conducted, with the efficacy outcome measured as the mean difference between baseline and post-programming atrioventricular synchrony. The results showed a statistically significant increase in atrioventricular synchrony by 11.3% (95% CI 7.0–15.7%; *p* < 0.01) in patients who underwent programming optimization (Figure 3). There was low heterogeneity between studies (*I*^2^ = 13%, *p* = 0.33). 

### 3.3. Cardiac Output

**Figure 4:**
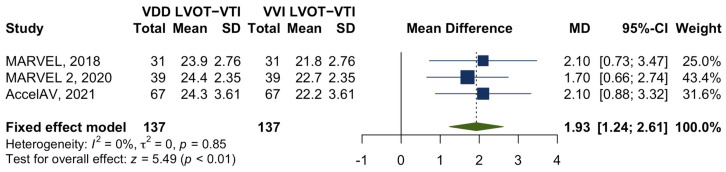
Forest plot of the change in LVOT-VTI comparing the VVI pacing mode with the VDD pacing mode. Abbreviation: LVOT-VTI = Left ventricular outflow tract velocity time integral [6,7,15].

Three studies involved one hundred thirty-seven patients who underwent echocardiogram procedures to assess the impact of leadless atrioventricular synchronous pacing on LVOT-VTI. All patients had a complete atrioventricular block and normal sinus function. Paired LVOT-VTI was obtained from echocardiograms, with the algorithm programmed to the VVI mode and VDD mode. A meta-analysis was undertaken to compare the changes in LVOT-VTI between the two pacing modes. It was found that leadless atrioventricular synchronous pacing could significantly increase the LVOT-VTI by 1.9 cm (95%CI 1.2–2.6, *p* < 0.01), without any heterogeneity observed across these studies (*I*^2^ = 0%, *p* = 0.85) (Figure 4).

### 3.4. Safety of Leadless Atrioventricular Synchronous Pacing

Seven studies reported safety endpoints following the implantation of atrioventricular synchronous leadless pacemakers. The follow-up duration of these studies varied from 0 to 12 months, with most studies having a relatively short follow-up duration (<3 months). In the 351 patients included in the studies, a sum of 22 complications related to the atrioventricular algorithm or procedures were reported, resulting in an overall complication rate of approximately 6.3%. However, only one study reported safety data beyond 1 year, and in this study of 32 patients, no ventricular pause or oversensing-induced tachycardia associated with the synchrony algorithm were observed [8]. In a study that involved patients undergoing transcatheter aortic valve implantation, two of the five patients examined displayed atrial under-sensing during a 1-month follow-up [16]. The remaining studies did not report any algorithm-related complications. Three studies [9,10,15] reported complications related to the procedure or device, including four pericardial effusions, eight cardiac rhythm disorders, one elevated threshold, one Micra-AV dislodgement, one death, and five others. There was a lack of safety data for follow-up periods longer than 1 year.

### 3.5. Sensitivity Analyses and Publication Bias

**Figure 5:**
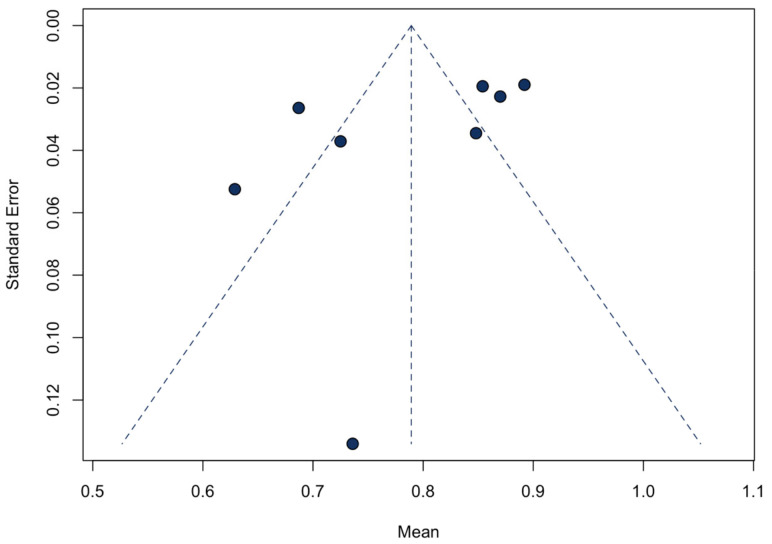
Funnel plot of the included studies demonstrating atrioventricular synchrony.

A sensitivity analysis of the primary efficacy endpoint was conducted. After the iterative omission of each included study, the pooled atrioventricular synchrony would not change (Supplementary Figure S1). The funnel plot for atrioventricular synchrony displayed symmetry (Figure 5), and the Egger’s test did not reveal a significant asymmetry (*p* = 0.16). Due to the small number of included studies for other outcomes, we did not construct funnel plots and conduct the Egger’s test for them.

## 4. Discussion

To our knowledge, this is the first systematic review and meta-analysis to examine the efficacy and safety of leadless pacemakers for atrioventricular synchronous pacing. All relevant data from 8 published studies with 464 participants were evaluated. The main findings can be summarized as follows. 

(1) Atrioventricular synchronous leadless pacemakers could contribute to a high atrioventricular synchrony, with a mean atrioventricular synchrony proportion of 78.9%. A regular programming optimization during follow-up was associated with a significant increase in atrioventricular synchrony. 

(2) Leadless pacemakers with the synchrony algorithm could significantly improve the ventricular performance, as measured by LVOT-VTI.

(3) The incidence of complications associated with leadless atrioventricular synchronous pacing was relatively low.

These findings demonstrated favorable efficacy and safety of this new technology, which could emerge as a potential alternative to conventional dual-chamber pacemaker implantation.

To date, the only leadless pacemaker device capable of atrioventricular synchronous pacing is the Micra-AV. The technical core for atrioventricular synchronous pacing consists of the innovative accelerometer-based algorithms. The Micra Accelerometer Sensor Sub-Study (MASS) and MASS2 studies were conducted to initially characterize the intracardiac accelerometer signals from the implanted Micra device [6]. In the accelerometer signal, four distinct segments corresponding to cardiac activity were observed: mitral/tricuspid valve closure (A1), aortic/pulmonic valve closure (A2), passive ventricular filling (A3), and atrial contraction (A4). Initially, the A1 and A2 signals were linked to ventricular events. Physicians could manually set appropriate blanking windows to reject the detection of the A1/A2 signals in the accelerometer and program a filtered and rectified accelerometer signal that exceeded the A3 threshold but was under the A4 threshold [17]. This allowed for the detection of the atrial contraction (A4) signal with an output of an atrial marker via telemetry. Then, a programmable atrioventricular interval was initiated, followed by ventricular contraction [17]. With this method, a typical cardiac cycle could finish, and atrioventricular synchronous pacing could be achieved. 

In the present study, a high proportion of atrioventricular synchrony at 78.9% was pooled from eight studies. Nevertheless, despite all studies reporting atrioventricular synchrony values, there were variations in the results. Out of the eight studies, only four had the main purpose of testing the safety and efficacy of this new technology [6,7,9,16], while the remaining four studies were designed to investigate the factors that promoted a higher atrioventricular synchrony [8,10,14,15]. Differences in the main endpoints between these studies may have led to a high heterogeneity. Additionally, there were substantial differences in patient selection, particularly in the proportion of patients with atrial fibrillation or atrial flutter. In a real-world study by Kowlgi et al. [9], 37% of the patients had atrial arrhythmias, compared to 7.5% in the MARVEL study [6]. In patients who developed atrial fibrillation or atrial flutter, irregular atrial contractions would bring about a drop in the A4 threshold and a worse sense of atrial mechanical activity, consequently impairing the atrioventricular synchrony. The most plausible indication for atrioventricular synchronous leadless pacemakers would be patients with high-grade or complete atrioventricular block without atrial arrhythmias [17]. According to our results, although Micra-AV did not achieve 100% atrioventricular synchrony similar to conventional transvenous VDD pacemakers [18], it still provided a viable option for patients who qualified for VDD pacing. 

The pacing burden also varied among the individuals who received atrioventricular synchronous leadless pacemakers, and those with an atrioventricular block typically had a higher pacing burden compared to those with a sinus node dysfunction. Studies by Arps et al. and Kowlgi et al. revealed that patients with a high pacing burden tended to exhibit a lower atrioventricular synchrony. This might be because their total atrioventricular synchrony relied more heavily on device-driven atrial tracking, in comparison with those with a high intrinsic atrioventricular synchrony. It is worth noting that a high VP% could increase the risk of pacing-induced cardiomyopathy, heart failure, and a worse clinical outcome [19,20]. Further studies are required to determine the optimal atrioventricular synchrony proportion to balance benefits and adverse effects.

Notably, our study also revealed that repetitive optimizations of the device programming after Micra-AV implantation could improve atrioventricular synchrony by 11.3%. This finding emphasizes the importance of post-implantation management and regular follow-up. Mitacchione et al. introduced a stepwise programming approach based on individual atrial electrical and mechanical characteristics to manage Micra-AV dyssynchrony [21]. Physicians could identify and address device issues by regularly programming parameters in the outpatient setting, which would offer a significant benefit to the patients. Several practical programming considerations for improving atrial sensing include adjusting the post-ventricular atrial blanking, the A3 timing window, the A3/A4 threshold, or other parameters [22]. 

The cardiac output represented as LOVT-VTI was significantly improved in the VDD pacing mode. This demonstrated a beneficial impact of leadless atrioventricular synchronous pacing on coordinated cardiac mechanical contractions, particularly for patients with a complete atrioventricular block. It is common that these patients have a lower cardiac output compared to those with a normal atrioventricular conduction, probably due to slow ventricular rates [23]. For this reason, those patients would experience palpitations, dizziness, syncope, or other discomforts. Micra-AV implantation could help achieve higher physiological atrioventricular synchrony and ventricular response in these patients. However, all three studies that reported LVOT-VTI had a short follow-up duration, and further research is required to determine the long-term change in cardiac output. In addition, while LVOT-VTI is a useful indicator, a long-term examination of the cardiac systolic function such as the ejection fraction is also necessary.

The current study summarized all adverse events reported in studies related to Micra-AV. The overall complication incidence associated with Micra-AV was 6.7%, which was lower than the typical complication incidence associated with transvenous pacemakers [24,25]. Most of those reported complications were linked to procedures, especially pericardial effusion, which is considered a potentially serious complication [26]. Complications such as ventricular pause or oversensing-induced tachycardia after Micra-AV implantation were deemed to be related to the synchrony algorithm. In all included studies, only two algorithm-related adverse complications were reported, indicating an extremely low incidence. Collectively, the present findings provide more reassuring information about the safety profile of leadless atrioventricular synchronous pacing and offer valuable reference information for its promotion.

Of note, a recent large-scale study revealed that leadless pacemakers significantly increased the risk of atrial fibrillation by 6.5% at 12-month postimplant follow-up [27]. This poor outcome might have been influenced by the decision to choose the VVI pacing mode in patients with sinus rhythm who could have potentially benefited from atrioventricular synchronous pacing. Atrial fibrillation developed more frequently in patients with VVI pacing mode than in those with DDD pacing mode, likely due to retrograde atrioventricular conduction, mitral regurgitation, adverse neuroendocrine reactions, and other factors associated with VVI pacing [28]. Patients with a high-degree atrioventricular block who receive atrioventricular synchronous leadless pacemakers may have a higher risk of atrial fibrillation due to a high expected pacing burden and a relatively low atrioventricular synchrony. Therefore, future studies are needed to improve the synchronous algorithms and achieve a better atrioventricular synchrony, and it is important to carefully screen appropriate candidates for leadless atrioventricular synchronous pacing in clinical practice.

The findings of our meta-analysis are encouraging. The innovative atrioventricular synchrony algorithm has significantly expanded the indications for leadless pacing and demonstrated a well-proven safety, promising to be an attractive alternative in various clinical settings. Nevertheless, it is important to note that this technology is still at an early stage. The existing studies were all conducted on a small scale and only focused on the short-term performance of this new device. Therefore, larger-sample studies with a long-term follow-up are necessary to confirm the overall benefits that leadless atrioventricular synchronous pacing may provide in the real-world population over time.

## 5. Limitations

The present meta-analysis has several limitations. Firstly, there is no randomized controlled study available yet, thus this study comprised only single-arm observational studies, which raises the possibility of a potential selection bias. Secondly, the approaches used to measure atrioventricular synchrony varied across studies, with some assessing the *p*-wave followed by a paced ventricle on surface electrocardiography, and others assessing “atrial mechanical sensed–ventricular pacing” detected at device interrogation. A case report showed that the former method was more reliable, whereas device interrogation might overestimate atrioventricular synchrony [21]. The differences in the measurement approaches in real-world settings might have introduced biases to our results. We further analyzed the indications and pacing burden in different studies to mitigate the biases. Thirdly, there was high heterogeneity for the pooled atrioventricular synchrony proportion, yet we conducted a meta-regression and did not perform further subgroup analysis due to an insufficient number of studies. Fourthly, we had to estimate and transform the data before combining them, as the included studies used different data representations. The estimated data were relatively unreliable, which might make the conclusions less definite. Finally, since leadless atrioventricular synchronous pacing is a relatively new technology, only a few small-sized studies met the inclusion criteria, limiting the strength of our conclusions.

## 6. Conclusions

The present meta-analysis demonstrates an excellent efficacy and safety of leadless atrioventricular synchronous pacing. This new device could improve atrioventricular synchrony and cardiac output in patients who are eligible for VDD pacing, with only a low complication incidence. However, since there are limited clinical data available, future large-scale and well-designed trials are still necessary to investigate the long-term performance of this novel technology and enable its broad implementation in clinical settings.

## Figures and Tables

**Table 1:** Characteristics of the studies included in the meta-analysis.

Study	Year	Single/Multicenter	Design	No. of Subjects	Study Quality
MARVEL [6]	2018	Multicenter	Prospective observational	70	Good
MARVEL 2 [7]	2020	Multicenter	Prospective observational	77	Good
Arps et al. [14]	2021	Single-center	Retrospective observational	50	Fair
Briongos et al. [8]	2022	Single-center	Prospective observational	32	Good
AccelAV [15]	2022	Multicenter	Prospective observational	152	Good
Kowlgi et al. [9]	2022	Single-center	Retrospective observational	43	Fair
Mechulan et al. [16]	2022	Single-center	Prospective observational	20	Fair
Neugebauer et al. [10]	2022	Single-center	Prospective observational	20	Good

**Table 2:** Baseline characteristics of the participants in the included studies.

Study	^a^ Age (y)	Female (%)	^b^ VP%	^a^ Estimated Atrioventricular Synchrony, %	^a^ Estimated LVOT-VTI (cm)		Complications
					VVI Mode	VDD Mode	
MARVEL [6]	71.3 ± 15.1	24(34.3)	NA	87.0 ± 18.2	21.8 ± 2.76	23.9 ± 2.76	None
MARVEL 2 [7]	77.6 ± 11.8	31(40.3)	NA	89.2 ± 12.0	22.7 ± 2.35	24.4 ± 2.35	None
Arps et al. [14]	69.0 ± 16.8	24(48.0)	10% (0, 92%)	84.8 ± 24.4	NA		NA
Briongos et al. [8]	78.0 ± 15.1	14(43.8)	NA	68.7 ± 14.7	NA		None
AccelAV [15]	77.2 ± 10.8	73(48.0)	^c^ 100% (99.7%, 100%)	85.4 ± 14.3	22.2 ± 3.61	24.3 ± 3.61	4 pericardial effusions, 4 cardiac rhythm disorder, 1 elevated threshold, 5 others
Kowlgi et al. [9]	76.4 ± 10.0	21(48.8)	AsVP ≥ 70%: 45.8 ± 46%; AsVP < 70%: 73.4 ± 34.6%	62.9 ± 34.4	NA		1 dislodgement
Mechulan et al. [16]	81.2 ± 6.8	5(25.0)	46.6 ± 40.1%	72.5 ± 8.3	NA		2 atrial under-sensing
Neugebauer et al. [10]	80.0 ± 8.0	11(55.0)	NA	73.6 ± 53.6	NA		4 atrial fibrillation, 1 death

Notes: ^a^ Values were estimated and transformed as mean ± SD, and the estimated data are displayed. ^b^ If individuals experienced multiple follow-ups, we extracted the VP% data of the first follow-up visit. Values are presented as mean ± SD or median with IQR. ^c^ This was calculated from 54 patients with complete atrioventricular block and normal sinus function. Abbreviations: AsVP = atrial synchronous ventricular pacing, LVOT = left ventricular outflow tract velocity time integral, NA = not available, VP% = percentage of ventricular pacing

## Data Availability

All data acquired that support the findings of this study are available on request from the corresponding author.

## Supplementary Materials

The following supporting information can be downloaded at: https://www.mdpi.com/article/10.3390/jcm12072512/s1, Method S1: Data Sources and Search strategies; Table S1. The quality assessment of included studies using the National Institutes of Health (NIH) quality assessment tool for before–after (pre-post) study with no control group; Figure S1: The sensitivity analysis of studies included for atrioventricular synchrony.
