# Supplementary material for: Efficacy and Safety of Leadless Pacemakers for Atrioventricular Synchronous Pacing: A Systematic Review and Meta-Analysis
Source: J Clin Med. 2023 Mar 27;12(7):2512. doi: 10.3390/jcm12072512 (PMC10095093; doi:10.3390/jcm12072512)

Supplementary Material

Efficacy and safety of leadless pacemakers for atrioventricular synchronous pacing: a systematic review and meta-analysis

Sijin Wu, Yuanhao Jin, Wenzhao Lu, Zhongli Chen, Yan Dai, and Keping Chen*

*** Correspondence:** Corresponding Author: chenkeping@263.net

1 Supplementary Method S1. Data Sources and Search strategies

Example of a search strategy run in Pubmed

#1
TS=((leadless[Title/Abstract]) OR (wireless[Title/Abstract])) OR (Micra[Title/Abstract])

#2
TS=((synchron*[Title/Abstract]) OR (atrioventricular[Title/Abstract])) OR (AV[Title/Abstract])

#3
TS=((pacemaker[Title/Abstract]) OR (pacing[Title/Abstract])) OR (artificial pacemaker[MeSH Terms])

#4
TS= #1 AND #2 AND #3

#5
TS= ("1948/01/01"[Date - Publication] : "2022/09/12"[Date - Publication])

#6
TS= #4 AND #5

2 Supplementary Table S1. The quality assessment of included studies using the National Institutes of Health (NIH) quality assessment tool for before–after (pre-post) study with no control group. (References [6–10,14–16] in reference list of the main text)

Study	Year	1	2	3	4	5	6	7	8	9	10	11	12	Quality
MARVEL [6]	2018	Y	Y	Y	NR	Y	Y	Y	CD	Y	Y	NR	NA	Good
Steinwender et al [7]	2020	Y	Y	Y	CD	CD	Y	Y	NR	Y	Y	NR	NA	Good
Arps et al [14]	2021	Y	Y	Y	NR	NR	Y	Y	NR	NR	Y	NR	NA	Fair
Briongos et al [8]	2022	Y	Y	Y	NR	NR	Y	Y	Y	Y	Y	Y	NA	Good
Chinitz et al [15]	2022	Y	Y	Y	CD	Y	Y	Y	CD	Y	Y	Y	NA	Good
Kowlgi et al [9]	2022	Y	CD	Y	NR	CD	Y	Y	NR	Y	Y	Y	NA	Fair
Mechulan et al [16]	2022	Y	Y	CD	Y	CD	Y	NR	Y	Y	NR	Y	NA	Fair
Neugebauer et al [10]	2022	Y	Y	Y	Y	CD	Y	Y	CD	Y	Y	Y	NA	Good

Abbreviations: CD, cannot determine; NA, not applicable; NR, not reported

3 **Supplementary Figure S1.** The sensitivity analysis of studies included for atrioventricular synchrony. (References [6–10,14–16] in reference list of the main text)

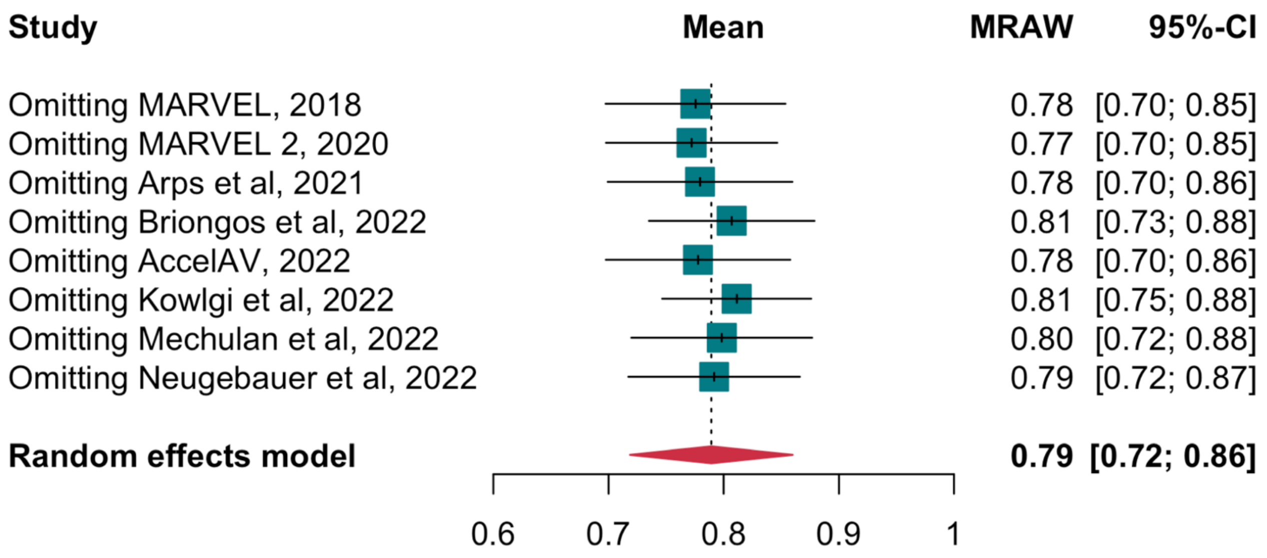

Supplement: Supplementary file 1 [file jcm-12-02512-s001.zip › jcm-2249694-supplementary.pdf]
